# Does fear of childbirth in nulliparous women affect fetal outcomes?

**DOI:** 10.1590/1806-9282.20251005

**Published:** 2025-12-05

**Authors:** Omer Demir, Gülsün Ozbay, Mirac Ozalp, Hidayet Sal, Gokce Omeroglu Kayıkcı, Emine Ahu Koç, Turhan Aran, Mehmet Armagan Osmanagaoglu

**Affiliations:** 1 Karadeniz Technical University, School of Medicine, Department of Gynecology and Obstetrics – Trabzon, Turkey.; 2 Medicana International Samsun Hospital, Department of Gynecology and Obstetrics – Samsun, Turkey.; 3 Liv Hospital Ulus, Department of Perinatology – Istanbul, Turkey.; 4 University of Health Sciences, Trabzon Kanuni Health Practice and Research Center, Department of Gynecology and Obstetrics – Trabzon, Turkey.; 5 Giresun University, School of Medicine, Department of Gynecology and Obstetrics – Giresun, Turkey.; 6 Zeynep Kamil Women and Children Diseases Training and Research Hospital, Department of Gynecology and Obstetrics – Istanbul, Turkey.

**Keywords:** Apgar score, Fear, Blood gas analysis, Pregnancy outcome, Tocophobia may prolong the duration of the second stage of labor; however, it does not affect fetal oxygenation, fetal cord blood gas parameters, or Apgar scores.

## Abstract

**OBJECTIVE:**

In this study, an investigation was undertaken to determine whether the well-being of the fetus is affected in pregnant women who have a severe fear of childbirth.

**METHODS:**

Participants were nulliparous pregnant women at 37 weeks of gestation. The Wijma Delivery Expectancy Questionnaire-A questionnaire, validated for the Turkish population, was used to assess the degree of fear of childbirth of the study subjects. Cases where labor began spontaneously and resulted in a vaginal delivery were included in the study. A total of 44 patients were investigated during the study period. The cases were divided into two groups according to the Wijma Delivery Expectancy Questionnaire-A scores.

**RESULTS:**

There were no statistically significant differences between the groups regarding fetal cord blood gas parameters and Apgar scores at the first and fifth minutes. Fear of childbirth in nulliparous cases did not affect the fetal cord blood gas parameters or Apgar scores. It was observed that the duration of the second stage of labor was statistically significantly longer in women with severe fear of birth (p=0.036).

**CONCLUSION:**

As a result, tocophobia can prolong the duration of the second stage of labor, does not affect fetal oxygenation, and does not change fetal cord blood gas parameters and Apgar scores. It is aimed to verify this result with large-scale studies and to encourage prospective studies.

## INTRODUCTION

Fear is a normal reaction to a perceived or existing danger. This reaction motivates people to warn against the danger and to act accordingly^1^. Since labor is an unpredictable process, the result is that many women experience fear of childbirth (FOC).

FOC is observed in most pregnant women, but its severity varies. Although pregnancy, birth, and postpartum processes result in relatively few complications today, many women experience fear of these processes^2-4^. It is acknowledged that an acceptable level of fear can help prepare the woman for birth^5,6^. However, if the FOC occurs before pregnancy or if this fear reaches very severe levels, it is referred to as "tocophobia"^5-7^. The term "tocophobia" was first used by Hofberg and Brockington^8^. Hofberg and Brockington described tocophobia as a form of anxiety specific to pregnancy, which results in a fear of death during childbirth. Tocophobia, in recent literature, exhibits the form of a significant fear of pathological birth.

It has been reported that 13% of women postpone pregnancy or refrain from conceiving due to FOC^6^. It has also been found that FOC can result in a decision to terminate a pregnancy^9,10^.

FOC brings with it various complications related to labor^11^. It activates various mechanisms that, in turn, activate the sympathetic nervous system and vasoconstriction in blood vessels^12^. The fearful and anxious woman during delivery releases increased levels of stress hormones such as catecholamines (adrenaline, noradrenaline)^5,13^. Catecholamines cause a decrease in blood flow to the uterus. A decrease in the amount of blood in the uterus causes a decrease in the oxygen level in the placental flow. As the oxygen to the fetus decreases, the fetus can become distressed. This situation increases the need for medical intervention for pregnant women^5^. Increased adrenaline resulting from anxiety causes a decrease in the amount of oxytocin that triggers contractions during delivery and can result in prolongation or even cessation of labor^5^. The second stage of birth is thereby prolonged. Prolonged labor increases the FOC in pregnant women. Communication problems between women who have FOC and birthing professionals can complicate the management of labor and delay clinical decision-making^13^.

Studies have shown that prenatal stress and anxiety are associated with low Apgar scores, intrauterine growth retardation, and low birth weight in the fetus^5,6,14^. However, it has been observed in the literature that this association is not based on fetal cord blood gas parameters and is based on Apgar scores, which are more objective and evaluatively dependent.

The leading and most accepted scale used in evaluating FOC is the Wijma Delivery Expectancy Questionnaire (W-DEQ), which was first described by Alehagen et al. in 2006^15^.

Therefore, in this study, a comparative evaluation of the W-DEQ scores and fetal cord blood gas parameters, which are more subjective criteria than the Apgar scores, was undertaken for the evaluation of fetal well-being.

## METHODS

This prospective study was performed at our outpatient clinic for pregnant women between 2019 and 2021, after obtaining ethical approval from the institutional review board (Karadeniz Technical University Faculty of Medicine Scientific Research Ethics Committee—2019/627) and informed consent from the patients. The study was created based on the principles set out in the Declaration of Helsinki.

The participants were nulliparous women who were at their 37th gestational week and had come for routine pregnancy examinations. The exclusion criteria were as follows: multiparity, a contraindicated condition for vaginal delivery; being in the active phase of labor; a maternal disease that could affect fetal cord blood gas parameters; fetal anomaly; need for induction of labor; delivery by cesarean section; and a history of psychiatric disorders while under medical care. After applying the exclusion criteria, 44 women were included in the study. The basic characteristics of the study population, such as education level, age, body mass index (BMI), birth week, baby weight, and gender of the baby, were recorded. BMI was calculated by dividing the participants’ weight (kg) by the square of their height (m^2^).

To assess the degree of FOC of the study subjects, the W-DEQ-A questionnaire validated for the Turkish population was used^16^. This form consists of 33 items in total, including complaints about FOC. It is a six-point Likert-type survey (0–5). While the minimum score on the scale is 0, the maximum score is 165. High scores indicate that the fear of birth experienced by women is high. The W-DEQ scores were collected in three subgroups. These groups were as follows: low birth fear (W-DEQ score between 0 and 65), moderate birth fear (W-DEQ score 66–84), and severe birth fear (W-DEQ score ≥85). In our study, we examined the patients in two groups: those with severe birth fear and those who did not, and we took the questionnaire score cut-off value as 85.

The W-DEQ-A form was completed by the assistant healthcare staff at 37 weeks of gestation. Subsequently, the cases where delivery commenced spontaneously and resulted in a vaginal delivery were included in the study. The patients were not given any training on birth and labor. In these cases, we examined whether there was a statistical relationship between the fetal cord blood gas parameters taken routinely at the end of delivery and the W-DEQ-A form results completed at 37 weeks of gestation.

The periods of the first and second stages of labor were recorded. The first stage of labor is the cervical opening stage and is called the total duration of the latent phase and the active phase. The second phase of labor begins when the cervical opening is completed and ends with the birth of the fetus.

### Statistical analysis

Statistical Package for the Social Sciences (SPSS) Version 20 was used for statistical analysis of the data. All data were placed in tables by giving the mean and standard deviation data. The Kolmogorov-Smirnov test was used to evaluate the conformity of the data to normal distribution. An independent t-test was used for comparative analysis of data conforming to normal distribution, and a Mann-Whitney U test was used for analysis of data not conforming to normal distribution. To assess the correlation between the population characteristics and neonatal well-being indicators with W-DEQ scores, the Spearman rho coefficients were calculated. p<0.05 was accepted as statistically significant.

## RESULTS

A total of 44 patients were analyzed during the study period. The cases were divided into two groups according to the W-DEQ-A scores. Accordingly, the group with a score ≥85, who experienced severe FOC, constituted Group 1, and those with a score<85, the group who did not exhibit a clinical FOC, formed Group 2. The basic characteristics of the study population are shown in Table 1. The mean ages of women in Group 1 and Group 2 were 27±3.8 (18–33) and 26.4±5.1 (19–37), respectively (p=0.415).

**Table 1:** The basic characteristics of the study population.

	Group 1 n=21	Group 2 n=23	p*
Age, years	27±3.8 (18–33)	26.4±5.1 (19–37)	0.415
BMI (kg/m^2^)	31.7±5.8	30.6±5.9	0.699
Education status	Middle school—1 High school—15 University—5	Primary school—4 Middle school—1 High school—10 University—8	0.132
W-DEQ-A score	90.9±7.5	69.9±5.6	**0.000**

BMI: body mass index; W-DEQ-A: Wijma Delivery Expectancy Questionnaire-A.

* <0.05. Bold values indicate statistically significant differences (p<0.05).

There were no statistically significant differences between the groups regarding fetal cord blood gas parameters and Apgar scores at the first and fifth minutes. The neonatal results of the two groups are shown in Table 2. A statistically significant difference was found between the two groups regarding birth weight (p=0.001). It was observed that the duration of the second stage of labor was statistically significantly longer in women with severe fear of birth (p=0.036).

**Table 2:** The neonatal results of the two groups.

	Group 1 n=21	Group 2 n=23	p*
Gestational week of birth, week	39 (37–41)	39 (37–41)	0.576
Duration of the first stage of labor, minutes	476.8±331.9 (120–1,140)	376.1±288.9 (78–1,080)	0.333
Duration of the second stage of labor, minutes	68.8±38.9 (9–180)	46.9±29.6 (9–120)	**0.036**
Weight of baby, kg	3,304.8±283	2,825.2±739.7	**0.001**
Height of baby, cm	50.2±1.7	48.8±2.9	0.144
Gender of baby (male/female)	15/6	11/12	0.136
Apgar at 1 min, median	7 (4–9)	8 (5–9)	0.131
Apgar at 5 min, median	9 (5–10)	9 (7–10)	0.401
pH value in cord blood gas	7.28±0.1	7.30±0.6	0.520
Base minus value in cord blood gas	-4.5±2.3	-4.2±2.6	0.606
Carbon dioxide value in cord blood gas	46.2±10.5	43±5.5	0.443
Oxygen value in cord blood gas	24.6±11.3	24.2±6	0.529
Bicarbonate value in cord blood gas	18.2±3.4	19.4±2.2	0.252

* <0.05. Bold values indicate statistically significant differences (p<0.05).

There were negative correlations between W-DEQ-A scores, first-minute Apgar scores, and fifth-minute Apgar scores, but these are not statistically significant (r: −0.190, p: 0.346; r: −0.081, p: 0.605, respectively). There was a significant positive correlation between W-DEQ-A scores and the weight value of the newborn (r: 0.437, p: 0.003) (Table 3).

**Table 3:** Spearman correlation analysis of population characteristics and neonatal well-being indicators with Wijma Delivery Expectancy Questionnaire scores.

	W-DEQ-A score (n=44)	
	R	p*
Age, years	-0.001	0.994
BMI (kg/m^2^)	-0.093	0.572
Gestational week of birth, week	-0.019	0.902
Duration of the first stage of labor, minutes	0.163	0.350
Duration of the second stage of labor, minutes	0.165	0.344
Weight of baby, kg	0.437	**0.003**
Height of baby, cm	0.293	0.054
Apgar at 1 min, median	-0.190	0.346
Apgar at 5 min, median	-0.081	0.605
pH value in cord blood gas	-0.146	0.356
Base minus value in cord blood gas	-0.179	0.257
Carbon dioxide value in cord blood gas	0.082	0.605
Oxygen value in cord blood gas	-0.086	0.586
Bicarbonate value in cord blood gas	-0.214	0.173

BMI: body mass index; W-DEQ-A: Wijma Delivery Expectancy Questionnaire-A.

* <0.05. Bold values indicate statistically significant differences (p<0.05).

## DISCUSSION

The unpredictable nature of childbirth makes an FOC reasonable to a certain extent^17^. It is estimated that 6–10% of all pregnant women experience a severe FOC^18^. Fear, which begins to increase as childbirth approaches, increases the levels of stress hormones and catecholamines in the body^19^. There is a significant amount of information in the literature regarding the effects of increased levels of catecholamines and cortisol hormones in maternal blood, which can trigger fetal distress^11^. In our study, it has been shown that this fear does not affect fetal cord blood gas parameters and Apgar scores in nulliparous women with clinical tocophobia. In one study that evaluated one hundred and one nulliparous pregnant women, it was reported that tocophobia negatively affects the outcomes of childbirth, and it was recommended that all pregnant women should be routinely screened before labor^20^. However, in the current study, tocophobia has not been identified as the cause of negative fetal outcomes.

In this study, there are several propositions to explain the condition of tocophobia and why it does not appear to affect fetal oxygenation and Apgar scores. First of all, it is known that increasing catecholamines in tocophobia decreases the blood flow to the uterus^12^. Although catecholamines prolong the labor, they do not affect fetal pH parameters, which may be attributed to the fact that catecholamine increases over a short period (during the labor process) and does not turn the oxygenation restriction into a chronic process^21^. In the current study, it was observed that the duration of the second stage of labor was statistically significantly longer in women with severe fear of birth. This is a consistent result with the studies in the literature^13,22^.

Catecholamines’ effect on pH parameters can explain the unaffected Apgar scores. Nevertheless, it is also believed that negativity is neutralized by the positive effect of cortisol, another hormone that increases with the stress brought on by tocophobia and has an effect on fetal lung maturity.

In the current study, it was seen that there was no statistical difference between the groups in terms of demographic data. In the literature, it was found that^22^ tocophobia was more severe, and W-DEQ-A scores were higher in younger patients. This result indicates that young nulliparous women do not know what to expect from the birthing process. In another study conducted by Rouhe et al. in 2009^18^, severe FOC was shown to be more common in young nulliparous women compared to multiparous women. In our study, unlike the literature, the average age of women who had severe FOC was not lower than other women.

Cord blood gas parameters and the Apgar scoring system are used to evaluate the well-being of the baby after birth^23^. These two assessment tools are also determinants of whether or not there has been fetal distress during delivery^24^. In the current study, negative correlations were found between the W-DEQ-A score and the first-minute Apgar score and the fifth-minute Apgar score in nulliparous women, but they were not statistically significant. When all cases were evaluated, a positive correlation was found between the W-DEQ-A score and birth weight. In numerous studies, it has been demonstrated that tocophobia prolongs birth time and can even change the mode of delivery to cesarean section^25^. It is suggested that this may affect Apgar scores in assessing fetal well-being. In this context, although the W-DEQ-A score had negative correlations relative to the Apgar value in the first minute and fifth minutes in the current study, the absence of statistical significance in the study groups shows that the severity of tocophobia in the subjects did not affect Apgar levels.

This research is the first study to investigate the effect of tocophobia on fetal cord blood gas parameters and Apgar scores in nulliparous women. The strength of this research is founded on the evaluation of women who have begun spontaneous vaginal delivery, thus ensuring that extraneous factors have not destroyed the reliability of the study. The limitation of the study is the relatively low number of cases.

## CONCLUSION

As a result, we observed that tocophobia can prolong the duration of the second stage of labor, does not affect fetal oxygenation, and does not change fetal cord blood gas parameters and Apgar scores. It is aimed to verify this result with large-scale studies and to encourage prospective studies.

## ETHICAL APPROVAL

Karadeniz Technical University Institutional Review Board gave approval for this study (Protocol Number 2019 / 627).

## Data Availability

The datasets generated and/or analyzed during the current study are available from the corresponding author upon reasonable request.
